# Complete mitochondrial genome and the phylogenetic position of the unicorn cod (*Bregmaceros macclellandi*)

**DOI:** 10.1080/23802359.2019.1682482

**Published:** 2019-11-05

**Authors:** Jianbiao Qiu, Yue Yu, Wei-Feng Chen, Ji-Nong Chen, Song Qin, Xin Peng

**Affiliations:** Zhejiang Mariculture Research Institute, Wenzhou, P. R. China

**Keywords:** *Bregmaceros macclellandii*, Gadiformes, mitogenome

## Abstract

The mitochondrial genome of *Bregmaceros macclellandii* was elucidated and analyzed for the first time in this study. It had a double-stranded DNA molecule with 17,319 bp in length and was made up of 37 genes (13 protein-coding genes, 2 rRNA genes, and 22 tRNA genes), and one control region. Phylogenetic analysis of 18 included species indicated that *B. macclellandii* was clustered closely with *Coelorinchus kishinouyei* and *Ventrifossa garmani*.

*Bregmaceros macclellandii* (Bregmacerotidae, Gadiformes), is a kind of brackish fish with the darkly pigmented pectoral fins (Akihisa et al. 2003; Riede 2004). For most adults, growing to 7 cm is their maximum (Bianchi 1985). It is normally distributed in the marine pelagic zone and its fitting region is subtropical. Since there is no economic interest, unicorn cod is usually thrown away once it is caught occasionally. The grown-up fishes seem to prefer mostly fish diet consisting of unicorn cod and others which belongs to the poor trophic level. It feeds on planktonic crustaceans (Reghu et al. 1996). In the present study, we have analyzed the mitochondrial genome information to comprehend its content, discerning the phylogenetic relationship of the fishes in Gadiformes and other orders.

The specimen of *B. macclellandii* was collected by a trawler in the South China Sea. DNA was extracted by the traditional phenol-chloroform method, ending with a final elution step in 100 μl. In this study, the complete mitochondrion genome of *B. macclellandii* was sequenced using Illumina Miseq. Excluding *B. macclellandii*, 16 species of other orders were selected to perform the phylogenetic tree which inferred from the maximum likelihood (ML) method and ML bootstrap analysis using PhyML3.0 with the AIC model (Guindon et al. 2010). The mitogenomic data was divided by two partitions: the first and second codons of the 12 protein-coding genes (except the light-strand encoded *ND6* gene). In addition, *Carapus bermudensis* and *Sirembo imberbis* are regarded as the outgroup.

The complete mitogenome of *B. macclellandii* is a double-stranded DNA molecule with the length of 17,319 bp and has been deposited in GenBank (accession no. MN067887), consisting of 13 protein-coding genes (PCGs), 2 ribosomal RNA genes (rRNA), 22 transfer RNA (tRNA) genes, and a control region (D-loop) with 1157 bp. The base composition of the whole mitogenome contains an A + T bias with an overall nucleotide composition of 29.85% T, 28.23% A, 27.54% C and 14.38% G. Furthermore, both the AT-skew and GC-skew are negative which are −0.028 and −0.314, respectively. For 13 PCGs, all of them initiated with a typical initiation codon ATG, while the most common termination codon is TAA (*nad1*, *nad2*, *cox1*, *atp8*, *atp6*, *nad4l*, *nad5*, and *nad6*), then is the incomplete termination codon TA- (*cox3*) or T– (*nad4*, *nad3*, *cox2*, and *cob*). Finally, it seems to have the gene recombination in the mitogenome with *trnA*, *trnN*, *trnC*, *trnS1*, *trnE*, *cob*, *trnT*, and *trnL2* genes inserted into *trnM* and *nad2*.

The phylogenetic tree divided the 18 fish species into five groups and all orders were monophyletic. Phylogenetic analysis showed that *B. macclellandii* clustered to *Coelorinchus kishinouyei* and *Ventrifossa garmani* which indicated the phylogenesis classification of *B. macclellandii* was consistent with the morphological result (Figure 1).

**Figure 1.**
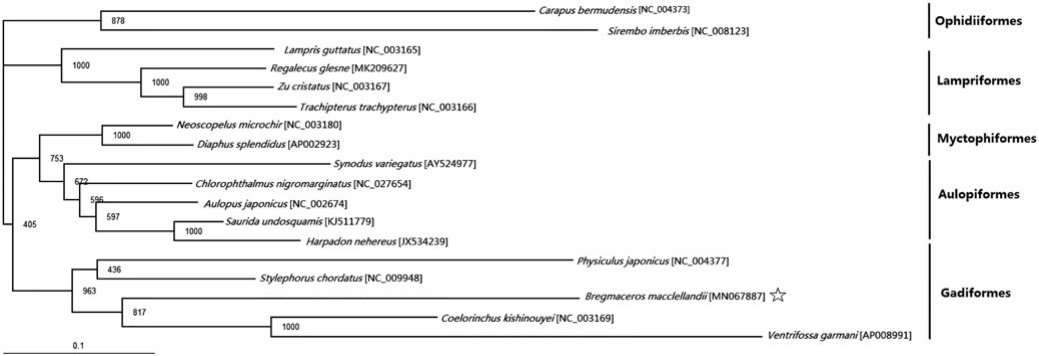
Phylogenetic position of *Bregmaceros macclellandii* phylogenetic relationships (maximum likelihood) of species of other orders based on the nucleotide sequence of 12 protein-coding genes in the mitochondrial genome. Numbers beside each node represent percentages of 1000 bootstrap values.
